# LncRNA Expression Profiles in Systemic Lupus Erythematosus and Rheumatoid Arthritis: Emerging Biomarkers and Therapeutic Targets

**DOI:** 10.3389/fimmu.2021.792884

**Published:** 2021-12-23

**Authors:** Han Wu, Shuxian Chen, Aifen Li, Kangyuan Shen, Shuting Wang, Sijie Wang, Ping Wu, Wenying Luo, Qingjun Pan

**Affiliations:** Clinical Research Center, Department of Clinical Laboratory, Key Laboratory of Prevention and Management of Chronic Kidney Disease of Zhanjiang City, Affiliated Hospital of Guangdong Medical University, Zhanjiang, China

**Keywords:** lncRNA, profile, systemic lupus erythematosus, rheumatoid arthritis, biomarker, therapeutic target

## Abstract

Systemic lupus erythematosus (SLE) and rheumatoid arthritis (RA) are two common multisystem autoimmune diseases that share, among others, many clinical manifestations and serological features. The role of long non-coding RNAs (lncRNAs) has been of particular interest in the pathogenesis of autoimmune diseases. Here, we aimed to summarize the roles of lncRNAs as emerging novel biomarkers and therapeutic targets in SLE and RA. We conducted a narrative review summarizing original articles on lncRNAs associated with SLE and RA, published until November 1, 2021. Based on the studies on lncRNA expression profiles in samples (including PBMCs, serum, and exosomes), it was noted that most of the current research is focused on investigating the regulatory mechanisms of these lncRNAs in SLE and/or RA. Several lncRNAs have been hypothesized to play key roles in these diseases. In SLE, lncRNAs such as GAS5, NEAT1, TUG1, linc0949, and linc0597 are dysregulated and may serve as emerging novel biomarkers and therapeutic targets. In RA, many validated lncRNAs, such as HOTAIR, GAS5, and HIX003209, have been identified as promising novel biomarkers for both diagnosis and treatment. The shared lncRNAs, for example, GAS5, may participate in SLE pathogenesis through the mitogen-activated protein kinase pathway and trigger the AMP-activated protein kinase pathway in RA. Here, we summarize the data on key lncRNAs that may drive the pathogenesis of SLE and RA and could potentially serve as emerging novel biomarkers and therapeutic targets in the coming future.

## 1 Introduction

Systemic lupus erythematosus (SLE) and rheumatoid arthritis (RA) are two common multisystem autoimmune diseases that share many clinical manifestations, serological profiles, immunological characteristics, and transcriptomes, for example shared type I interferon (IFN)-stimulated genes of peripheral blood mononuclear cell (PBMC) transcriptomes (1). Furthermore, the co-occurrence of SLE and RA within the same person or within members of a nuclear family indicates that they shared common etiological factors (2–4). In addition to the traditional treatment options with hormones and immunosuppressants (5, 6), a large variety of biological drugs is now available for the treatment of SLE and RA (7–9), however, the clinical response and functional remission rate of these drugs are still not satisfactory. Therefore, treatment strategies for SLE and RA need further improvement by adopting different approaches (9, 10).

In the human genome, 98% of the products are non-coding RNAs (11), and those with a size length greater than 200 nucleotides (NT) are defined as long non-coding RNAs (lncRNAs) (12). LncRNAs have poor protein-coding potential (13–15), except for certain micropeptides or polypeptides that can perform specific biological functions (16). It is well known that the regulation of gene expression *via* lncRNAs occurs mainly through variable interactions with DNA, RNA, and proteins (17, 18), and are thus involved in a variety of important regulatory processes, such as the silencing of the X-chromosome, chromatin modifications, transcriptional activation interference, and post-transcriptional modifications (19). The role of lncRNAs is of particular interest in the pathogenesis of autoimmune diseases (20, 21). They could participate in inflammatory pathways in autoimmune diseases and promote the release of inflammatory factors such as TNF-α, IL-6 (22), IL-8, IL-1β (23), IFN-I (24) to aggravate or alleviate diseases. In addition, lncRNAs are widely found in many bodily fluids and are highly stable in the plasma, potentially serving as biomarkers for multiple diseases (25).

Here, we aimed to summarize the roles of lncRNAs as emerging novel biomarkers and therapeutic targets in SLE and RA. We conducted a narrative review and summarized original articles on lncRNAs associated with SLE and RA patients, published until November 1, 2021.

## 2 LncRNAs and SLE

It is well known that genetic and environmental risk factors are key players involved in the pathogenesis of SLE (26, 27), and the multi-organ involvement, highly heterogenic clinical characters, and differences in the degree of severity lead to major challenges in its diagnosis and treatment (28–30). Recently, increasing evidence shows that many lncRNAs are dysregulated and may have a key role in the development of SLE (31). Transcriptome sequencing results revealed a large number of novel lncRNAs in PBMC, serum and exosomes of SLE patients and animal models. Their potential use as biomarkers and their correlation with clinical features were also studied. Studies focusing on the expression profiles of novel lncRNAs in PBMCs and serum from SLE patients and animal models revealed their potential as biomarkers as well as regulatory mechanisms in SLE.

### 2.1 The Expression Profiles of LncRNAs in the PBMCs of SLE

Recently, lncRNAs derived from PBMCs of patients with SLE have been a research hotspot because of their large presence and rich variety. Abnormal numbers and functions of PBMCs are significantly related to SLE pathogenesis (32, 33). A study showed that 137 lncRNAs-derived from PBMC were identified as differentially expressed in normal controls (n=15) and SLE patients (n=15) *via* microarray technology, with 83 upregulated and 54 downregulated lncRNAs. Among them, two lncRNAs, ENST00000604411.1 and ENST00000501122.2, were significantly upregulated, while another two, lnc-HSFY2-3:3 and lnc-SERPINB9-1:2, were significantly downregulated in patients with SLE. The study showed that the upregulated ENST00000604411.1 could lead to X chromosome inactivation by protecting the active-X from ectopic silencing, and thus playing a pathogenic role in SLE (34). In addition, the levels of the two upregulated lncRNAs were positively correlated with the clinical activity index (SLEDAI score) of SLE patients (ENST00000604411.1 (r=0.593, P=0.020), ENST00000501122.2 (r=0.539, P=0.038), suggesting that the levels of these two lncRNAs could be used to evaluate the disease activity in SLE patients (34). LncRNA TCONS_00483150 in PBMCs was significantly decreased in patients with SLE compared with health controls, and its expression was significantly correlated with anti-Rib-P autoantibody, which may be anovel biomarker for the diagnosis of SLE (35). It has also been reported that lncRNAs taurine-upregulated gene 1 (TUG1), linc0949, nuclear-enriched abundant transcript 1 (NEAT1), and linc0597 were expressed at lower levels in the PBMCs of SLE patients (31, 36, 37). Among them, TUG1 was further reduced in patients with lupus nephritis, and its expression was negatively correlated with the SLEDAI score (r=0.904, P< 0.001). NEAT1 is known as an early lipopolysaccharide (LPS) response lncRNA that can modulate the innate immune response *via* the toll-like receptor (TLR) signaling pathway (38, 39). In addition, the levels of NEAT1 expression in PBMCs of SLE patients was significantly increased and was positively correlated with the disease activity. Furthermore, NEAT1 was found to affect the expression of inflammatory chemokines and cytokines by activating the late mitogen-activated protein kinase (MAPK) signaling pathway, which could regulate the immune response of T and B cells, and participate in the development of SLE, thus providing a potential therapeutic target for SLE (37). Another study showed that the up-regulated NEAT1 was negatively correlated with Th1/Th2 balance, which might affect the occurrence and progression of SLE (40). Hence, lncRNAs NEAT1, linc0949, and linc0597 are expected to be promising diagnostic markers for SLE, whereas TUG1 is expected to be a clinical diagnosis and disease activity marker.

Additionally, for the expression of lncRNAs in PBMCs, lnc5150 was lower in patients with SLE (n=76) than in healthy controls (n=71) (41). The expression of lncRNA AC007278.2 was high in SLE patients and could modulate the expression of inflammatory chemokines and cytokines. The study showed that ACC007278.2 could promote B cell maturation by down-regulating its target gene *CCR7* and T follicular helper cells, participating in SLE. Therefore, AC007278.2 may be used as a molecular biomarker for the diagnosis and treatment of SLE (42). Compared to healthy controls, metastasis-associated lung adenocarcinoma transcript 1 (MALAT-1), which is mainly expressed in human monocytes, was significantly increased in SLE patients, and could modulate the silent mating type information regulation 2 homolog 1 (SIRT1) pathway directly (43). Another study reported that MALAT1 also could participate in type I interferon-mediated SLE by up-regulating OAS2, OAS3 and OAS-like (OASL) in CD4^+^ T cells (24).

LncRNA growth arrest-specific 5 (GAS5) regulates growth arrest, apoptosis, cell cycle, and replication in T cell lines and non-transformed lymphocytes (44). GAS5 was reported to be related with an increased risk of development of SLE in a murine model (45). Also, GAS5 has been found to be involved in disease progression in SLE patients (46) and may be involved in the development of SLE *via* the MAPK signaling pathway (25). These results indicate that PBMC-derived lncRNAs may play a vital role in the pathogenesis of SLE, but the specific mechanisms remain unclear.

Recently, the genetic significance of lncRNAs in many autoimmune diseases has been investigated, and most of the susceptibility loci for SLE were found to be located in noncoding regions of the genome (47, 48). A novel SLE susceptibility locus in a lncRNA gene (SLEAR) was identified at the single-nucleotide polymorphism rs13259960, which can result in decreased SLEAR production in PBMCs from patients with SLE. Moreover, it could interact with RNA binding proteins and thus affect the downstream target genes. In addition, the level of SLEAR expression was correlated with the percentage of PBMC death in patients with SLE (47). The rs145204276 ID/DD genotypes in the promoter region of the LncRNA-GAS5 gene may have a protective effect against SLE by up-regulating LncRNA-GAS5 expression and its targets miR-21 and phosphatase and tensin homolog deleted on chromosome 10 (PTEN) (48). Two functional promoter variants in linc00513, significantly overexpressed in SLE, were reported to be possible candidates in promoting genetic susceptibility to SLE (49). Till now, these studies are very few, and several still need large-scale data verification to provide novel insights into the genetics of SLE.

Abnormal proliferation and activation of B cells can produce large quantities of autoantibodies, which are deposited in the kidney and other tissues, further inducing inflammation and tissue damage. This is considered the core of the pathogenesis of SLE (50, 51). Among all SLE treatments targeting B cells, belimumab is the only biological agent approved by the FDA (52). Recently, Dimitrioset et al. reported that CD19-targeted chimeric antigen receptor (CAR) T-cell therapy was successful in refractory SLE, and the rapid disappearance of dsDNA autoantibodies during CD19 CAR-T cell therapy suggested CD19-targeted plasmablasts as the major source of these antibodies (53).

The activation of type I interferon (IFN-I) in B cells is also closely related to the pathogenesis of SLE (54, 55). Recently, based on this theory, SLE treatment has mainly focused on blocking IFN-1 or its receptor (56), or targeting improved B cell survival to reduce the level of immunoglobulin G (IgG) autoantibodies (57–59). It has been reported that myeloid-derived granulocyte suppressor cells (G-MDSCs) promote B cell IFN-1 signal activation in lupus MRL/LPR mice (60). TLRs or interferon-α (IFN-α) can induce the expression of B cell activating factor (BAFF) (61, 62). LncRNA NEAT1 was highly expressed in G-MDSCs of lupus MRL/LPR mice, and G-MDSCs enhanced TLRs or IFN-α to produce BAFF (60). Furthermore, BAFF enhanced the activation of B cell IFN-1 signaling by inhibiting the expression of cytokine signal transduction inhibitor 3, which is involved in the occurrence and development of SLE. NEAT1 deficiency alleviated the symptoms of lupus and inhibited the activation of IFN-1 signaling in B cells of pristane-induced lupus mice, indicating that lncRNA NEAT1 plays a key role in the activation of B cell IFN-1 signaling pathway (60). LincRNA00892 also has been reported possibly activated CD4^+^ T and B cells by targeting heterogeneous nuclear ribonucleoprotein K (hnRNP K) and subsequently up-regulating the expression of CD40L, thereby playing a pathogenic role in SLE (63). These data suggest that lncRNA is involved in modulating B cell activation and the production of autoantibodies, thus providing a new theory and intervention strategy for SLE.

Accumulating evidences have demonstrated that T cells are central in the pathogenesis of SLE (64, 65). LncRNAs uc001ykl.1 and ENST00000448942 in T cells from SLE patients (n=24) were downregulated compared to normal controls (n=21), and their expression was correlated with the erythrocyte sedimentation rate (ESR) (66). LncRNA GAS5 has been reported to possibly upregulate the adenovirus E4 promoter-binding protein (E4BP4) by inhibiting miR-92a-3p, attenuating the self-reactivity of CD4^+^ T cells in SLE, playing a protective role in SLE (67). Therefore, targeting lncRNAs expressed in T cells and their signaling pathways may be a potential therapy for SLE.

### 2.2 The Expression Profiles of LncRNAs in the Serum and Plasma of SLE

LncRNAs are stable in serum and plasma and may serve as novel non-invasive biomarkers for SLE (68). The expression of linc-DC and GAS5 has been found to be decreased in the plasma of SLE patients (n=163) compared with health controls (n=80), while linc0597 is increased (68). Another study identified 1873 lncRNAs derived from the plasma of SLE patients through gene ontology analysis, with 221 upregulated and 1652 downregulated lncRNAs (lg|FC| ≥ 2.0 and P ≤ 0.05), of which Yippee-like-4 (YPEL4) was related to the receptor immunoglobulin G (FcγR) pathway (69). The FcγR mediates the interaction between immune complexes and immune cells and participates in the activation and regulation of a variety of immune responses, which play important roles in humoral immunity and cellular immunity. The combination of FcγR and the IgG Fc segment could stimulate immune cells to release inflammatory mediators, activate CD4^+^ and CD8^+^ T cells, and amplify humoral and cellular immunity, thereby promoting the pathogenesis of SLE (70). However, the molecular mechanism of action has not yet been identified. In another study, compared with the normal control group, 1315 significantly differentially expressed lncRNAs (lg|FC| ≥ 2.0 and P ≤ 0.05) were found in the plasma of SLE patients (n=24) (68), with significantly increased levels of linc0597, lnc0640, and lnc5150 and significantly decreased levels of GAS5 and lnc7074. However, the molecular mechanism of action has not yet been identified. These lncRNAs may be involved in the regulation of the MAPK signaling pathway, promoting the inflammatory response in SLE, and could be used as novel potential diagnostic biomarkers (68). This panel of five lncRNAs (linc0597, lnc0640, lnc5150, GAS5, lnc7074) had a high accuracy for the diagnosis of SLE (AUC=0.966), and could also be used to distinguish SLE from RA patients (AUC=0.683 and 0.910, respectively) (25). Subsequently, in the external validation phase, the expression levels of these five lncRNAs were investigated in thirty RA patients and thirty-one SLE patients. The results showed that the levels of GAS5 and linc0597 were significantly lower in SLE patients in the testing group than in RA patients, while no significant differences were found in the levels of lnc7074, lnc-DC, lnc0640, and lnc5150 between the two groups, which may be different from other autoimmune diseases (Sjogren’s syndrome) (25). Finally, the co-expression analysis found that GAS5, lnc0640 and lnc5150 may be involved in the pathogenesis of SLE *via* the MAPK signaling pathway. The competitive endogenous RNA (ceRNA) network showed that the forementioned five lncRNAs bind competitively with miRNAs and regulate the expression of their target genes, hence their aberrant expression may have a vital role in SLE pathogenesis. Therefore, it is hypothesized that analyzing the ceRNA network in SLE may help expand the understanding of transcriptomes (especially non-coding transcriptomes) and improve the understanding of the pathogenesis, diagnosis, and treatment of SLE (71, 72).

### 2.3 The Expression Profiles of LncRNAs in the Exosomes of SLE

Exosomes are endocytic membrane-derived vesicles, measuring 30–120 nm in length, and participate in the communication among cells and in the delivery of contents (e.g., proteins, lipids, nucleic acids) to target cells (73–75). Evidence indicates that exosomal non-coding RNAs play a vital role in the pathogenesis of autoimmune diseases, such as SLE and RA (76, 77).

With recent research findings, the role of lncRNAs in SLE has gradually become clear. The abnormal expression of lncRNAs in patients with SLE can be used as a potential biomarker to assist in SLE diagnosis and treatment. However, the specific mechanisms need to be confirmed. In addition, more evidence is needed to investigate the other roles of lncRNA in SLE, such as whether it is related to clinical features, diagnosis, and prognosis, and whether it can be used to evaluate the clinical treatment effect on SLE. These findings will provide novel ideas and directions for lncRNA research.

## 3 LncRNA and RA

RA is a typical chronic systemic autoimmune disease dominated by inflammatory synovitis. Genetics, smoking, air pollution, and gender are all considered risk factors for RA (78, 79). Its pathogenesis is complex, and pro-inflammatory factors such as interleukin (IL)-1, IL-17, IL-22, tumor necrosis factor alpha (TNF-α), IL-6, and matrix metalloproteinase (MMP) have been confirmed to be related to the development of RA (80–84). Recently, emerging studies have found that lncRNAs play a critical role in the pathogenesis of RA (85–88). In addition, many lncRNA disorders are related to RA disease activity, indicating that the role of lncRNA is conducive to the clinical diagnosis of RA and may serve as a new target for its treatment.

### 3.1 The Expression Profiles of LncRNAs in the PBMCs of RA

Studies have shown that lncRNA HOTAIR derived from both serum and PBMCs is significant highly expressed in RA and could be used as a novel biomarker for its diagnosis (89, 90). In addition, it may also play a vital role in RA pathogenesis. The expression of HOTAIR in chondrocytes stimulated by LPS was significantly reduced. Overexpression of HOTAIR reduced the rate of LPS-induced cell proliferation and inhibited inflammatory cytokine (IL-17, IL-23) production. The overexpression of HOTAIR also inhibited the activation of nuclear factor kappa B (NF-κB) in chondrocytes stimulated by LPS by blocking p65 nuclear transport, resulting in the reduction of IL-1β and TNF production (91). This suggests that regulating the expression of HOTAIR may be a potential treatment strategy for RA.

The lncRNA GAS5 is related to several autoimmune diseases. The expression of GAS5 in PBMCs and fibroblast-like synoviocytes (FLS) is lower in the serum of patients with RA (n=35) than that in normal controls (n=35) (92, 93). Moreover, GAS5 can be used as a ceRNA to directly target miR-222-3p, upregulate the expression level of Sirt1, and inhibit the proliferation and inflammation of RA-FLS. It is also reported that the overexpression of lncRNA GAS5 in the PBMCs of patients with RA can activate the AMP-activated protein kinase (AMPK) pathway, negatively regulate the expression of IL-6 and IL-17, and alleviate RA disease activity (94). These findings suggest that GAS5 activation is a potential target for RA treatment. Compared with healthy controls (n=20), the expression of lncRNAs MIR22HG and ENST00000619282 is significantly increased, while the expression of lncRNAs down syndrome critical region (DSCR9), LINC01189 and MAPKAPK5-AS1 is significantly decreased in PBMCs from patients with RA (n=20). According to gene ontology analysis, these significantly altered lncRNAs are mainly involved in the regulation of autophagy and apoptosis (95).

Some lncRNAs can act as ceRNAs to regulate miRNA function and are involved in RA progression (96). Compared with normal controls (n=40), the expression level of lncRNA HIX003209 in the PBMCs of patients with RA (n=43) was higher and positively correlated with the expression levels of TLR2 and TLR4 in macrophages (97). Further studies have found that HIX003209 can reversibly promote the proliferation and activation of macrophages by modulating the inhibitory effect of the κBα (IκBα)/NF-κB signaling pathway. In contrast, HIX003209 can act as a ceRNA to participate in TLR4-mediated inflammatory responses by binding to miR-6089 in macrophages (98). This suggests that the HIX003209-miR-6089-TLR4 signaling pathway may be a novel target for the treatment of RA.

### 3.2 The Expression Profiles of LncRNAs in the FLS of RA

The FLS is a key effector cell type responsible for the inflammation of the synovium and destruction of bone and cartilage. It can mediate the production of inflammatory mediators and matrix degrading enzymes and play a critical role in the occurrence and development of RA (99–101).

In the synovial tissue of patients with RA (n=30), a total of 349 lncRNAs were significantly upregulated, and 806 were significantly downregulated (lg|FC| ≥ 2.0 and P ≤ 0.05) compared with those in the normal control group (n=30). Among these lncRNAs, the levels of lnc-AL928768.3 and lnc-AC091493.1 expression were positively correlated with the RA-DAS28 score and the level of CRP, which is considered to be a novel diagnostic marker and activity index of RA. These lncRNAs can regulate their target mRNAs [e.g., Syndecan 1 (SDC1), leukotriene B4 (LTB_4_)], and are thus implicated in the abnormal immune response of RA and in promoting the proliferation of FLS *via* multiple pathways (102). In terms of promoting RA inflammation, the level of lncRNA Fer-1-like family member 4 (FER1L4) in FLS and synovial tissues (STs) of patients with RA was low, whereas NLR family CARD domain containing 5 (NLRC5) was highly expressed (103). NLRC5 promotes RA progression by modulating the NF-κB signaling pathway (104). In contrast, overexpression of FER1L4 reduced the expression of NLRC5 and inflammatory factors. This suggests that FER1L4 may be a potential therapeutic target for RA (105). LncRNA linc00152 was reported to be up-regulated in RA-FLS, which could promote TAK1 expression by targeting miR-103a and thus activate the NF-κB pathway. Also, transcription factor Ying Yang 1 (YY1) could also directly promote linc00152 expression, thus forming a linc00152/NF-κB feedback loop that could promote RA-FLS inflammation (106). LncRNA FOXD2 adjacent opposite strand RNA 1 (FOXD2-AS1) was found to promote the proliferation and invasion of RA-FLS by regulating the miR-331-3p/PIAS3 pathway (107). LncRNA LERFS (lowly expressed in rheumatoid fibroblast-like synoviocytes) could promote synovial aggression and joint destruction by interacting with heterogeneous nuclear ribonucleoprotein Q (hnRNP Q) (108). LncRNA ZNF667-AS1 was reported to be down-regulated in RA-FLS, and its overexpression could play a protective role in RA by sponging miR-523-3p, thus inactivating the JAK/STAT signaling pathway (109). The down-regulated expression of the lncRNA X-inactive specific transcript (XIST) was found to inhibit the proliferation of synovial fibroblasts (SFs) by promoting the miR-126-3p/NF-κB pathway, thereby playing a protective role in RA (110). Therefore, targeting these lncRNAs in the FLS of RA may be used as a new strategy for RA therapy.

Comparing the expression profile of FLS-derived lncRNAs from patients with RA and healthy controls, p38 inhibited cutaneous squamous cell carcinoma associated lincRNA (lncRNA PICSAR) was found to be highly expressed in the FLS and synovial fluid of patients with RA. When PICSAR small-interfering RNA was used to reduce the expression of PICSAR, the levels of IL-6, IL-8, and MMP-3 were significantly reduced. Thus, PICSAR may be act as the ceRNA of miR-4701-5p and then promote the proliferation, invasion, and migration of RA FLS (111).

*In vitro*, overexpression of lncRNA maternally expressed gene 3 (MEG3) reversed both the high expression of miR-141 in LPS-stimulated chondrocytes and the production of IL-23. In animal experiments, overexpression of lncRNA MEG3 inhibited the protein kinase B (PKB; also known as AKT) and mammalian target of rapamycin (mTOR) (AKT/mTOR) signaling pathway. This suggests that lncRNA MEG3 can also be used as a ceRNA to inhibit inflammation by downregulating miR-141 and AKT/mTOR signaling pathways (112). In addition, in a CFA-induced rat RA model, MEG3 was low in synovial tissue and FLS, while the level of NLRC5 was increased, suggesting that MEG3 may potentially regulate the progression of RA by targeting NLRC5 (113).

LncRNA-H19 is highly expressed in the FLS of patients with RA (114). In a collagen-induced arthritis (CIA) mouse model, the expression of lncRNA-H19 was closely associated with the proliferation of synovial cells, and knocking down lncRNA-H19 could inhibit the proliferation of MH7A human synovial cells. LncRNA-H19 can act as a ceRNA of miR-124a to inhibit the expression of CDK-2 and MCP-1 (115, 116). As already known, miR-124A may participate in the pathogenesis of RA through several molecular mechanisms. miR-124A can suppress the proliferation and inflammation of RA-FLS by targeting the phosphatidylinositol 3-kinase (PI3K)/NF-κB pathway (117). The methylation of miR-124a helps attenuate IL-1β-mediated RA-FLS proliferation and the expression of TNF-α (118). Also, miR-124a was found to inhibit the proliferation and invasion of RASFs by decreasing the expression of MMP3/13 and IL-1 (119). It has also been reported that the expression of lncRNA-H19 was inhibited by liver X receptor (LXR) agonists, suggesting that LXR may have an anti-arthritis function (120). Therefore, targeting the lncRNA-H19 and its downstream signaling pathway or using LXR agonists may be new strategies for RA treatment.

In addition, many other lncRNAs have been reported to be involved in the pathogenesis of RA. Overexpression of lncRNA zinc finger antisense 1 (ZFAS1) was found to upregulate miR-27a, and thereby promote the migration and invasion ability of RA-FLS, suggesting a pathogenic role of ZFAS1 in RA (121). Low expression of lncRNA intersectin1-2 (ITSN1-2) inhibits the nucleotide-binding oligomerization domain 2 and receptor-interacting protein 2 (NOD2/RIP2) signaling pathway and reduces the proliferation and inflammation of RA-FLS (122). Overexpression of the lncRNA downregulated in liver cancer stem cells (DILC) can induce FLS apoptosis and downregulate the expression of IL-6, thereby reducing RA inflammation (123). Increased expression of lncRNA RP11-83J16.1 in FLSs from RA patients has been identified, which could regulate the levels of the frequently rearranged in advanced T cell lymphomas-1 (FRAT1) and β-catenin expression and thus promote cell proliferation, migration, invasion, and decreased apoptosis in RA-FLS (124). Compared with healthy controls (n=40), the expression of lncRNA PlncRNA-1 was downregulated in the serum and fibroblasts of active RA patients (persistent symptoms) (n=34), but not in inactive RA patients (long term of no or few symptoms after active RA) (n=36). In addition, PlncRNA-1 plays a central role in RA possibly by regulating on TGF-β1 expression (125). In summary, these lncRNAs may act as therapeutic targets for RA.

### 3.3 The Expression Profiles of LncRNAs in the Exosomes of RA

Recently, lncRNAs have been found to be enriched in exosomes (126), which can be released by almost all cells, and are present in bodily fluids, thus making them attractive targets for biomarker research (127). LncRNA NEAT1 was reported to be highly expressed in RA and PBMC-derived exosomes in patients with RA (n=5), that could contribute to the pathogenesis of RA through the delivery of lncRNA NEAT1. Furthermore, the study also highlighted that lncRNA NEAT1 shuttled by PBMC-derived exosomes plays a critical role in the development of RA by regulating the miR-23a/MDM2/SIRT6 axis (128). Subsequent studies have also shown that, compared with the exosomes from normal controls (n=20), there was a significant increase in the expression of NEAT1 in the exosomes of patients with RA (n=68). Also, NEAT1 might act as a ceRNA for miR144-3p to restrict its function, and thus increase the expression of the miR144-3p-targeted gene (Rho associated coiled-coil containing protein kinase 2, ROCK2) in CD4^+^ T cells, promoting the progression of RA (129). Another study showed that the levels of a set of lncRNAs, HOTAIR, Luca-15 Specific Transcript (LUST), anti-NOS2A, MEG, TUG1, NEAT1, Small Nucleolar RNA Host Gene 4 (SNHG4), Highly Accelerated Region 1B (HAR1B), and GAS5, have higher expression levels in seral exosomes of patients with RA (n=28) than in the seral exosomes of normal controls (n=10) (89). Hence, these molecules are likely to serve as biomarkers for RA. However, nowadays, little is known about the exact downstream signaling pathways of exosomal lncRNAs in modulating inflammatory response and autoimmunity. Further studies are warranted to fill this research gap.

## 4 The Similarities and Differences in lncRNAs Between SLE and RA

Studies showed that some lncRNAs can regulate both SLE and RA, but the mechanisms involved are different. For example, GAS5 may participate in the pathogenesis of SLE through the MAPK pathway, but it regulates the progression of RA by activate the AMPK pathway (25, 94). Overexpressed NEAT1 in the G-MDSCs from the lupus murine model could lead to BAFF secretion and thus promote the activation of B cells so as to accelerate the progression of SLE, while the delivery of NEAT1 by PBMCs-derived exosomes could promote the development and progression of RA *via* the microRNA-23a/MDM2/SIRT6 axis (60, 128). However, apart from these similarities and differences in lncRNAs between SLE and RA, their function and molecular mechanisms are still not well understood. Although both diseases are closely related to autoimmune inflammation, different organs are involved in the pathogenesis of SLE and RA; in SLE kidneys, blood cells, skin, brain, heart, lungs, and joints are mainly affected (21), while RA commonly affects the joints in the hands, wrists, knees, etc. (130). Therefore, further studies are needed to reveal the similarities and differences between lncRNAs in SLE, RA, and also other autoimmune diseases. The lncRNAs implicated in SLE and RA are shown in **Table 1**. 

**Table 1:** LncRNAs implicated in SLE and RA.

LncRNAs	Site	Expression	Signaling	References
**SLE**				
GAS5*	PBMC/Serum	DOWN	MAPK signaling pathway	(25)
NEAT1*	PBMC	UP	MAPK signaling pathway	(37)
TUG1*	PBMC	DOWN	Unknown	(36)
ENST00000604411.1	PBMC	UP	X chromosome inactivation	(34)
ENST00000501122.2	PBMC	UP	Unknown	(34)
TCONS_00483150	PBMC	DOWN	Unknown	(35)
lnc5150	PBMC/Serum	DOWN	MAPK signaling pathway	(25)
AC007278.2	PBMC	UP	Unknown	(42)
MALAT-1	PBMC	UP	SIRT1 signaling pathway	(43)
uc001ykl.1	B cell	DOWN	Unknown	(66)
ENST00000448942	B cell	DOWN	Unknown	(66)
YPEL4	Serum	UP	FcγR pathway	(69)
linc0949	PBMC	DOWN	Unknown	(31)
linc0597	PBMC/Serum	DOWN	Unknown	(31)
lnc0640	Serum	UP	MAPK signaling pathway	(68)
lnc7074	Serum	DOWN	MAPK signaling pathway	(68)
linc-DC	Serum	UP	Unknown	(68)
**RA**				
HOTAIR	Chondrocytes	DOWN	NF-κB signaling	(91)
HOTAIR	PBMC/Serum exosomes	UP	Unknown	(89, 90)
GAS5*	PBMC/Serum/S-erum exosomes	UP	AMPK pathway	(89)
GAS5*	FLS	DOWN	SIRT1 signaling pathway	(94)
MIR22HG	PBMC	UP	Unknown	(95)
ENST00000619282	PBMC	UP	Unknown	(95)
DSCR9	PBMC	DOWN	Unknown	(95)
LINC01189	PBMC	DOWN	Unknown	(95)
MAPKAPK5-AS1	PBMC	DOWN	Unknown	(95)
HIX003209	PBMC	UP	IκBα/NF-κB/HIX003209-miR-6089-TLR4	(98)
lnc-AL928768.3	STs	UP	Unknown	(102)
lnc-AC091493.1	STs	UP	Unknown	(102)
FER1L4	FLS/STs	DOWN	NF-κB signaling	(103, 104)
linc00152	FLS	UP	NF-κB signaling	(106)
FOXD2-AS1	Serum/STs	UP	miR-331-3p/PIAS3 pathway	(107)
LERFS	FLS	DOWN	Unknown	(108)
ZNF667-AS1	FLS	DOWN	JAK/STAT signaling	(109)
XIST	FLS	DOWN	miR-126-3p/NF-κB signaling	(110)
PICSAR	FLS	UP	Unknown	(111)
MEG3	Chondrocytes	UP	AKT/mTOR	(112)
lncRNA-H19	FLS	UP	PIK3/NF-κB pathway	(114, 117)
ZFAS1	FLS	UP	Unknown	(121)
ITSN1-2	FLS	DOWN	NOD2/RIP2	(122)
RP11-83J16.1	FLS	UP	Unknown	(124)
NEAT1*	PBMC exosomes	UP	Unknown	(129)
LUST	Serum exosomes	UP	Unknown	(89)
anti-NOS2A	Serum exosomes	UP	Unknown	(89)
SNHG4	Serum exosomes	UP	Unknown	(89)
HAR1B	Serum exosomes	UP	Unknown	(89)
TUG1*	Serum exosomes	UP	Unknown	(89)

DOWN is downregulated, UP is upregulated. The lncRNAs marked with * are shared both SLE and RA.

## 5 Future Perspectives

Recently, the studies focusing on investigating the role of lncRNAs in autoimmune diseases have significantly increased. However, the current studies are mainly focused on the possible role of lncRNAs as biomarkers, by screening their expression profiles in diagnostic data or by monitoring the activity of autoimmune diseases. Conversely, information on the role of their biological function and molecular mechanisms is still relatively scarce.

**Figure 1:**
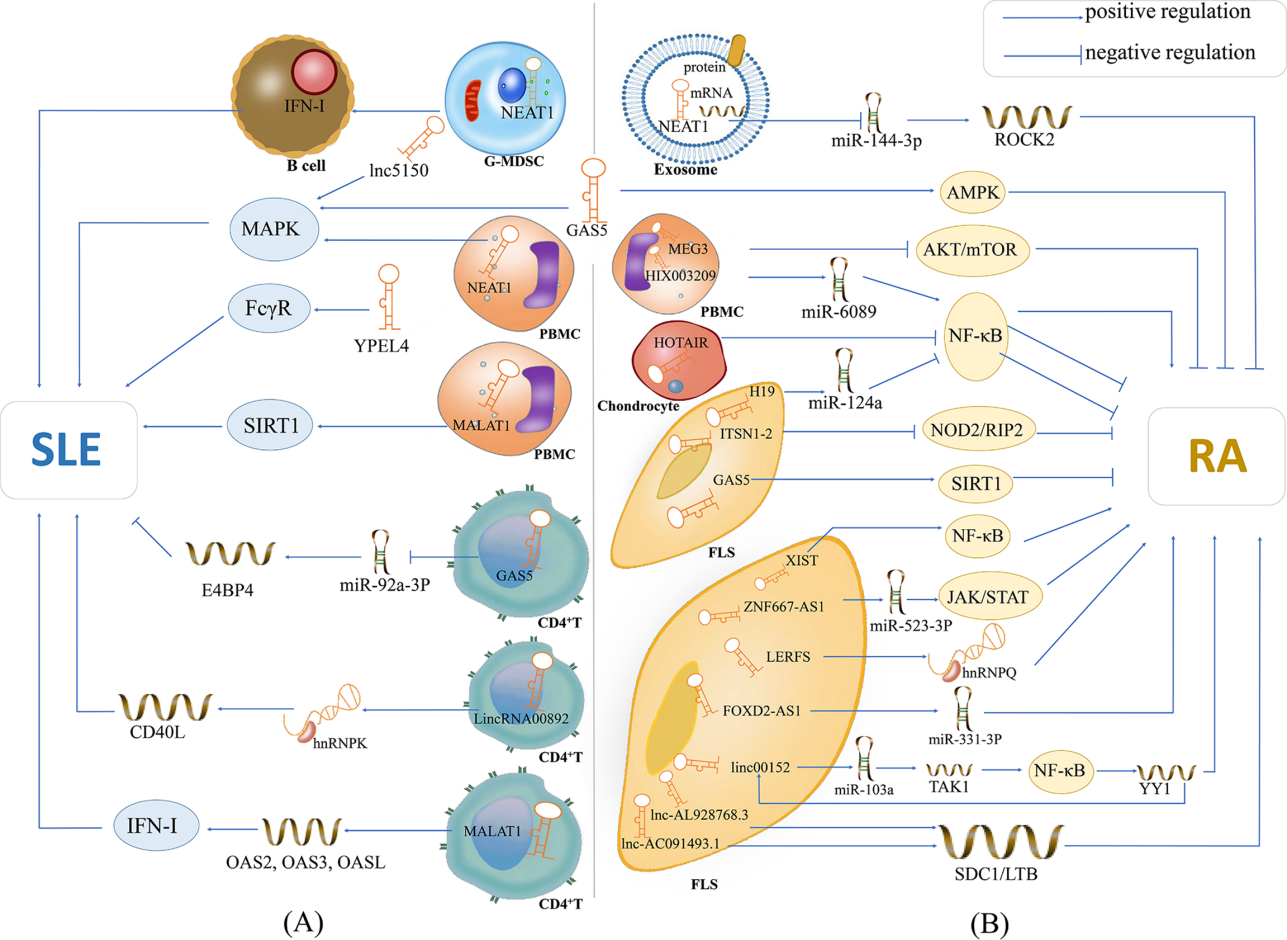
The potential mechanisms of lncRNAs in SLE and RA. **(A)** lncRNA NEAT1 is overexpressed in G-MDSCs and induces the promotion of G-MDSCs on IFN-I signaling activation of B cells, contributing to the pathogenesis of SLE; lnc5150 and GAS5 in serum participate in the regulation of the MAPK signaling pathway, and promote the inflammatory response of SLE; lncRNA YPEL4 in serum promotes the onset of SLE through FcγR-mediated phagocytosis; lncRNA NEAT1 in PBMCs affects the expression of inflammatory mediators through activating the MAPK signaling pathway; lncRNA MALAT1 is overexpressed in PBMCs and can modulate the SIRT1 pathway directly, then promote the inflammatory response of SLE; lncRNA GAS5 in CD4^+^ T cells can upregulate E4BP4 by inhibiting miR-92a-3p and attenuating the self-reactivity of CD4^+^ T cells, playing a protective role in SLE; LincRNA00892 can activate CD4^+^ T by targeting hnRNP K and subsequently up-regulating the expression of CD40L, thereby playing a pathogenic role in SLE; lncRNA MALAT1 in CD4^+^ T cells can participate in type I interferon-mediated SLE by up-regulating OAS2, OAS3 and OASL. **(B)** lncRNA NEAT1 shuttled by PBMC-derived exosomes plays critical role in the development of RA by acting as a ceRNA for miR144-3p to restrict its function, and thus increase the expression of the miR144-3p-targeted gene ROCK2; lncRNA GAS5 in the serum of patients with RA activates the AMPK pathway; lncRNA MEG3 acts as ceRNA to inhibit inflammation by down-regulating AKT/mTOR signaling pathways; lncRNA HIX003209 in LPS-treated chondrocytes promotes the proliferation and activation of macrophages by modulating the inhibitory effect of the IκBα/NF-κB signaling pathway; lncRNA HOTAIR inhibits the activation of NF-κB in chondrocytes and reduce inflammation of RA; lncRNA-H19 acts as the ceRNA of miR-124a to inhibit the proliferation and invasion of RASF; lncRNA ITSN1-2 inhibits the NOD2/RIP2 signaling pathway and reduces the proliferation and inflammation of RA-FLS; GAS5 in FLS acts as a ceRNA to directly target miR-222-3p, upregulates the expression of Sirt1 and inhibits the proliferation and inflammation of RA; lncRNA XIST can inhibit the proliferation of SFs by promotion of of miR-126-3p/NF-κB pathway, thereby playing a protective role in RA; lncRNA ZNF667-AS1 is overpressed in RA-FLS, which plays a protective role in RA by sponging miR-523-3p and thus inactivation of JAK/STAT signaling pathway; LncRNA LERFS is lowly expressed in RA-FLS and can promote synovial aggression and joint destruction by interacting with hnRNP Q; lncRNA FOXD2-AS1 can promote the proliferation and invasion of RA-FLS through regulating the miR-331-3p/PIAS3 pathway; lncRNA linc00152 is up-regulate in RA-FLS, which can promote TAK1 expression by targeting miR-103a and thus activate the NF-κB pathway; lncRNA AL928768.3 and lnc-AC091493.1 can regulate their target mRNAs (e.g., SDC1, LTB), and thus implicate in the abnormal immune response of RA or promote the proliferation of FLS *via* multiple pathways in patients with RA. Also, transcription factor YY1 can promote linc00152 expression directly, and thus forming a linc00152/NF-κB feedback loop, which can promote RA-FLS inflammation.

In addition to being potential biomarkers in SLE and RA, lncRNAs were found to participate in the modulation of the inflammatory and autoimmune responses, which are shown in **Figure 1**. However, the upstream regulatory mechanism of the abnormal expression of these lncRNAs in SLE and RA is still unclear, and there is a lack of studies addressing such question. Moreover, the downstream regulatory mechanism of these lncRNAs in SLE and RA still needs further investigation. These studies may greatly improve our understanding of the pathogenesis of human autoimmunity and provide novel therapies for autoimmune diseases.

## Author Contributions

HW, SXC, AFL, KYS, STW and SJW wrote the manuscript and designed the figure. PW, WL, and QP revised the manuscript. All authors contributed to the article and approved the submitted version.

## Funding

This study was supported by the National Natural Science Foundation of China (no. 82070757), the Project of “Dengfeng Plan” from Affiliated Hospital of Guangdong Medical University and Affiliated Hospital of Guangdong Medical University “Clinical Medicine+” CnTech Co-construction Platform, and Guangdong Basic and Applied Basic Research Foundation (no. 2019A1515012203), the Zhanjiang City Program for Tackling Key Problems in Science and Technology (no. 2019B01179).

## Conflict of Interest

The authors declare that the research was conducted in the absence of any commercial or financial relationships that could be construed as a potential conflict of interest.

## Publisher’s Note

All claims expressed in this article are solely those of the authors and do not necessarily represent those of their affiliated organizations, or those of the publisher, the editors and the reviewers. Any product that may be evaluated in this article, or claim that may be made by its manufacturer, is not guaranteed or endorsed by the publisher.

## Abbreviations

AKT/mTOR, protein kinase B and mammalian target of rapamycin; AMPK, AMP-activated protein kinase; ceRNA, competitive endogenous RNA; DSCR9, down syndrome critical region; FcγR, receptor of immunoglobulin G; FLS, fibroblast-like synoviocytes; FOXD2-AS1, FOXD2 adjacent opposite strand RNA 1; FRAT1, the frequently rearranged in advanced T cell lymphomas-1; GAS5, growth arrest-specific 5; G-MDSCs, myeloid-derived granulocyte suppressor cells; hnRNP K, heterogeneous nuclear ribonucleoprotein K; hnRNP Q, heterogeneous nuclear ribonucleoprotein Q; IFN-I, type I interferon; ITSN1-2, intersectin1‐2; IκBα, inhibitory κBα; LTB_4_, leukotriene B4; lncRNA, long non-coding RNA; MALAT-1, metastasis associated lung adenocarcinoma transcript 1; MAPK, mitogen-activated protein kinase; MEG3; maternally expressed gene 3; NEAT1, nuclear-enriched abundant transcript 1; NF-κB, nuclear factor kappa B; NOD2/RIP2, the nucleotide-binding oligomerization domain 2; PBMC, peripheral blood mononuclear cell; PTEN, phosphatase and tensin homolog deleted on chromosome 10; RA, rheumatoid arthritis; RASF, rheumatoid arthritis synovial fibroblasts; ROCK2, Rho associated coiled-coil containing protein kinase 2; SIRT1, silent mating type information regulation 2 homolog 1; SLE, systemic lupus erythematosus; SDC1, Syndecan 1; Tfh cells, T follicular helper cells; YPEL4, Yippee-like-4; YY1, Ying Yang 1.
